# Lactate Metabolism Subtypes Analysis Reveals CCDC80 as a Novel Prognostic Biomarker in Gastric Cancer

**DOI:** 10.7150/jca.97640

**Published:** 2024-08-26

**Authors:** Xiang Li, Yaqi Du

**Affiliations:** 1 Department of Gastroenterology, The First Hospital of China Medical University, Shenyang 110000, China.; 2 Department of Medical Oncology, the First Hospital of China Medical University, Shenyang 110000, China.

**Keywords:** lactate metabolism, unsupervised clustering, gastric cancer, CCDC80, drug sensitivity

## Abstract

Lactate metabolism plays a vital role in tumor progression. Currently, gastric cancer (GC) has a poor prognosis. Therefore, our research aimed to investigate novel biomarkers related to lactate metabolism in patients. Patient data from The Cancer Genome Atlas (TCGA) and Gene Expression Omnibus (GEO) database were divided into subtypes based on the expression of lactate metabolism-related genes (LMRGs). Based on the subtypes, we identified coiled-coil domain containing 80 (*CCDC80*) for further investigation. Univariate and multivariate Cox regression models were constructed to determine the prognostic value of *CCDC80* in GC. We further explored the mechanism by which *CCDC80* affects GC prognosis using gene set enrichment analysis (GSEA). Immune infiltration and drug sensitivity analyses were also performed. Finally, immunohistochemical staining was used to evaluate CCDC80 expression in normal and tumor tissues. We observed that CCDC80 was overexpressed in GC samples and was significantly associated with T and pathological stages. Multivariate Cox analysis identified high *CCDC80* expression as an independent prognostic marker. GSEA indicated that the oxidative phosphorylation pathway was highly enriched in the low *CCDC80* expression group. Moreover, *CCDC80* was associated with immune cell infiltration, especially that of M2 macrophages. Patients with higher *CCDC80* expression exhibited lower sensitivity to paclitaxel. In conclusion, our findings demonstrate that *CCDC80* is a critical regulator in GC progression and immune response and is associated with lactate metabolism, and it could be used as a novel biomarker for prognostic and chemotherapy treatment purposes.

## Introduction

According to the 2020 worldwide cancer statistics, gastric cancer (GC) is the fourth leading cause of cancer-related deaths worldwide 1. The main treatment methods for GC include surgery, chemotherapy, and targeted therapy. However, the prognosis of GC remains poor, especially for advanced GC, with a 5-year survival rate of < 10% 2, 3. Multiple factors contribute to this result, including the difficulty in early diagnosis and treatment, drug resistance, a high rate of recurrence, and tumor heterogeneity. Thus, identifying novel molecular biomarkers that can predict clinical prognosis would help further understand the pathogenesis of GC and promote personalized treatments for GC.

One promising area of research in cancer biology is the investigation of metabolic reprogramming, specifically focusing on lactate metabolism, which has been implicated in various aspects of tumor progression, including metastasis 4 and immune evasion 5, 6. The alteration of energy metabolism in tumor cells is a hallmark of cancer 7. Tumor cells use glucose through glycolysis and produce lactic acid even under aerobic conditions; the process is termed aerobic glycolysis and is also known as the Warburg effect 8, 9. This phenomenon contributes to tumor development and drug resistance 10, 11; however, the mechanisms underlying aerobic glycolysis are unclear.

CCDC80 (Coiled-Coil Domain Containing 80, also called DRO1, URB, CL2) is an extracellular matrix protein and was first found to be upregulated in the brown adipose tissue of mice with mild obesity 12. In obese patients, the level of circulating CCDC80 protein is related to fatty liver disease, insulin secretion, and atherosclerosis 13. *CCDC80* is also related to several cancers and serves as a tumor suppressor in colorectal cancer 14, pancreatic cancer 15, thyroid cancer 16, and malignant melanoma 17. However, the association of *CCDC80* with GC, particularly its relationship with lactate metabolism, immune cell infiltration, drug sensitivity, and clinical outcomes, has not yet been reported.

In this study, we sought to identify novel prognostic markers for GC related to lactate metabolism. To that aim, we broadly divided patients with GC into three subgroups based on unsupervised clustering of lactate metabolism-related genes (LMRGs) and further explored the function of each subgroup. *CCDC80* was screened as a differentially expressed gene (DEG) among these three subgroups and validated as a predictive biomarker of survival ability and drug sensitivity.

## Materials and Methods

### Acquisition and preparation of genetic and clinical data

RNA sequencing and clinical data of GC patients were downloaded from The Cancer Genome Atlas (TCGA) data repository (https://portal.gdc.cancer.gov) using the TCGAbiolinks package 18. This included clinical cases which had pathological TNM staging and survival information.

Three microarray datasets, containing GSE62254 19, GSE15459 20, and GSE57303 21, were downloaded from Gene Expression Omnibus (GEO) database (https://www.ncbi.nlm.nih.gov/geo/) using the GEOquery package 22, and all samples were from Homo sapiens. After background-corrected and normalization, we got three GEO expression datasets, respectively. Batch effects from these independent datasets were corrected by using the sva R package.

### Download of LMRGs and unsupervised clustering

We acquired LMRGs from the Molecular Signatures Database (MSigDB; https://www.gsea-msigdb.org/gsea/msigdb/index.jsp) 23. In this study, five datasets named respectively HP lactic acidosis, HP increased serum lactate, HP severe lactic acidosis, HP lactic aciduria, and GOBP lactate metabolic process were used in the presented analyses.

Survminer and survival R packages (https://CRAN.R-project.org/package=survival) were used to perform survival analysis for further screening LMRGs that could potentially affect prognosis of patients with GC. Based on these genes, samples from TCGA and GEO cohorts were separated into distinct molecular subtypes by the unsupervised clustering method. We do this by using the Bioconductor ConsensusClusterPlus package 24. Consensus clustering was conducted by hierarchical clustering algorithm for 1000 iterations to ensure the stability of classification.

We calculated the ESTIMATE score, immune score, and stromal score for each sample using the estimate R package 25 to estimate immune and stromal infiltration, and analyzed the differences in each score among the clusters.

A gene set variation analysis (GSVA) analysis of the expression matrix was per-formed by the GSVA package 26, using 18 common biological functional pathway genes as a reference gene set. We further analyzed the main function of each cluster depending on the difference of pathways enrichment in subgroups.

### Identification of *CCDC80* and correlation analysis with lactate metabolism

The DEGs were screened by the limma package 27, and the volcanic maps of DEGs were drawn by the ggplot2 package (https://ggplot2.tidyverse.org) to show the differential expression of DEGs. DEGs satisfies p.value < 0.05 and | logFC | > 0.7. We conducted DEGs analysis among Cluster3 and Cluster4, Cluster3 and Cluster1+2, Cluster1+2+3 and Cluster4, respectively. Venn diagram was generated and displayed the intersection of DEGs in three groups.

We confirmed *CCDC80* by survival analysis of intersecting genes among DEGs. Survival curves were constructed using the survival R package. To verify the clinical significance of *CCDC80*, independent t-tests were used to determine differences in the expression of *CCDC80* in samples with different clinical characteristics in the TCGA dataset.

We analyzed the expression of CCDC80 in different subtypes. In order to clarify the correlation between CCDC80 and lactate metabolism, we analyzed the correlation between CCDC80 and the mRNA expression of hypoxia-inducible factor 1α (HIF -1α), Lactate dehydrogenase A (LDHA), and Lactate dehydrogenase B (LDHB). In addition, we also analyzed the correlation of *CCDC80* with some other lactate metabolism-related genes.

### Multivariate Cox regression of CCDC80 and nomogram model

To estimate the hazard ratio (HR) of OS, we conducted univariate and multivariate Cox regression analysis. We analyzed the prognosis classification of risk score by multivariate Cox regression analysis by timeROC R package 28, and prognostic classification efficacy for 1-, 3-, and 5-year were displayed. With the result we had gotten from multivariate Cox analysis, the nomogram which can show the survival probability for 1-, 3-, and 5-year was built with the rms R package (https://CRAN.R-project.org/package=rms).

### Pathway enrichment analysis

We set the median of *CCDC80* expression as cut-off and divided the patients with GC into two groups: low and high *CCDC80* expression groups. Limma package was used to identify DEGs between two groups, with the threshold of log fold change |logFC| > 0.6.

The potential biological mechanisms and pathways of DEGs were explored with the clusterProfiler package 29 in R to annotate and visualize the Gene ontology (GO) analysis and Kyoto Encyclopedia of Genes and Genomes (KEGG) pathways.

Gene enrichment analysis was also performed using gene set enrichment analysis (GSEA). The h.all.v7.2.symbols.gmt dataset in the MSigDB of the GSEA website was selected as the reference gene set to assess the influences of different expressions on each reference set. GSEA was carried out by the method of default weighted enrichment statis-tics, and the number of random combinations was designed to be 1,000 times.

### Immune cell infiltration analysis

We use the CIBERSORTx website (https://cibersortx.stanford.edu/) 30 to estimate the immune infiltrate in each sample and calculate the relationship between *CCDC80* and immune cell subsets. Immune cell infiltration analysis was also done by GSVA R package with the method of single-sample gene set enrichment analysis (ssGSEA) to explore the difference of immune cell infiltration between *CCDC80* low and high expression groups. Subsequently, we conducted a correlation analysis between *CCDC80* and immune cells in TIMER2.0 (http://timer.cistrome.org/) 31.

IOBR R package 32 was used to calculate the immune cell fraction in the *CCDC80* low and high expression groups by the method of quanTIseq. In addition, we also assessed the differences of important signatures between groups.

### Drug sensitivity analysis

The GDSC database (www.cancerrxgene.org/) 33 can be used to find tumor drug response data and sensitive markers of the genome. We download drug response data from the GDSC resource. The pRRophetic algorithm is used to construct a ridge regression model according to gene expression, and then we use IC50 to predict the sensitivity of low and high *CCDC80* expression groups to common anticancer drugs.

### Somatic variant analysis

The gene mutations in GC patients were shown by the maftools R package 34, and the total number of non-synonymous mutations per trillion bases could be used to calculate the tumor mutation load (TMB) in the low and high *CCDC80* expression groups.

### The clinical validation cohort collection

We collected primary tumor samples from the First Hospital of China Medical University between 2010-2016, which consisted of 80 GC patients' tumor specimens and adjacent non-tumor tissue specimens. Study protocols were approved by the Ethics Committee of The First Hospital of China Medical University (AF-SOP-07-1.1-01). All participants provided written informed consent. Patients diagnosed with GC without other serious diseases were enrolled in the study. During surgery, tumor tissue (within 3 cm of the tumor edge) and normal gastric tissue (3 cm from the tumor edge) were collected from the 80 patients and stored at -80 °C for future use. The inclusion criteria were used as follows: (1) patients pathologically confirmed with gastric cancer; (2) patients subjected to surgery; (3) patients aged 18-80 years. The exclusion criteria included receiving neoadjuvant chemotherapy or radiotherapy, remnant gastric cancer, and postoperative death within 3 months. The pathological diagnoses and classifications were estimated according to the AJCC Cancer Staging Manual (7th edition) 35. Survival follow-up data were noted by telephone or medical records.

### Immunohistochemistry and evaluation with clinicopathologic characteristics

Immunohistochemistry of CCDC80 was performed on the validation cohort. All tissue specimens were fixed in neutral formaldehyde, embedded in paraffin, and sectioned (thickness, 4μm). The streptavidin-peroxidase immunohistochemical method was used to enhance staining intensity. Tissue sections were incubated at 4 °C overnight with An-ti-CCDC80 (1:200) (ab224050; rabbit anti-human, polyclonal, Abcam, MA, USA). Then, samples were lightly counterstained with hematoxylin, dehydrated in alcohol, and mounted. Two pathologists, blinded to the clinical data, independently scored the slides in each sample by evaluating the staining intensity and percentage of stained cells in representative areas. The slides were analyzed by standard light microscopy.

The staining intensity was scored as 0 (negative), 1 (weak), 2 (moderate), or 3 (strong). The percentage of cells stained was scored as 1 (1-25%), 2 (26-50%), 3 (51-75%), or 4 (76-100%). A final combined score of 0-12 was obtained by multiplying the intensity and percentage scores. Specimens with scores ≥ 4 were considered CCDC80-positive, and those with scores < 4 indicated CCDC80-negative.

We also further explored the prognostic value of CCDC80 protein expression and the correlation between CCDC80 protein expression groups and clinicopathologic characteristics of patients with GC.

### Statistical analysis

Survival curves were constructed using the R package survival. GSEA was used to identify the pathways that were significantly enriched in low and high *CCDC80* expression groups. Group comparisons were performed on continuous variables. Independent t-tests were used for normally distributed variables, and Mann-Whitney U tests were conducted on other variables. All statistical tests were two-sided, and *p* < 0.05 was considered statistically significant. Statistical analyses were performed using R software (version 4.1.1, www.r-project.org).

## Results

### Data processing

**Figure 1:**
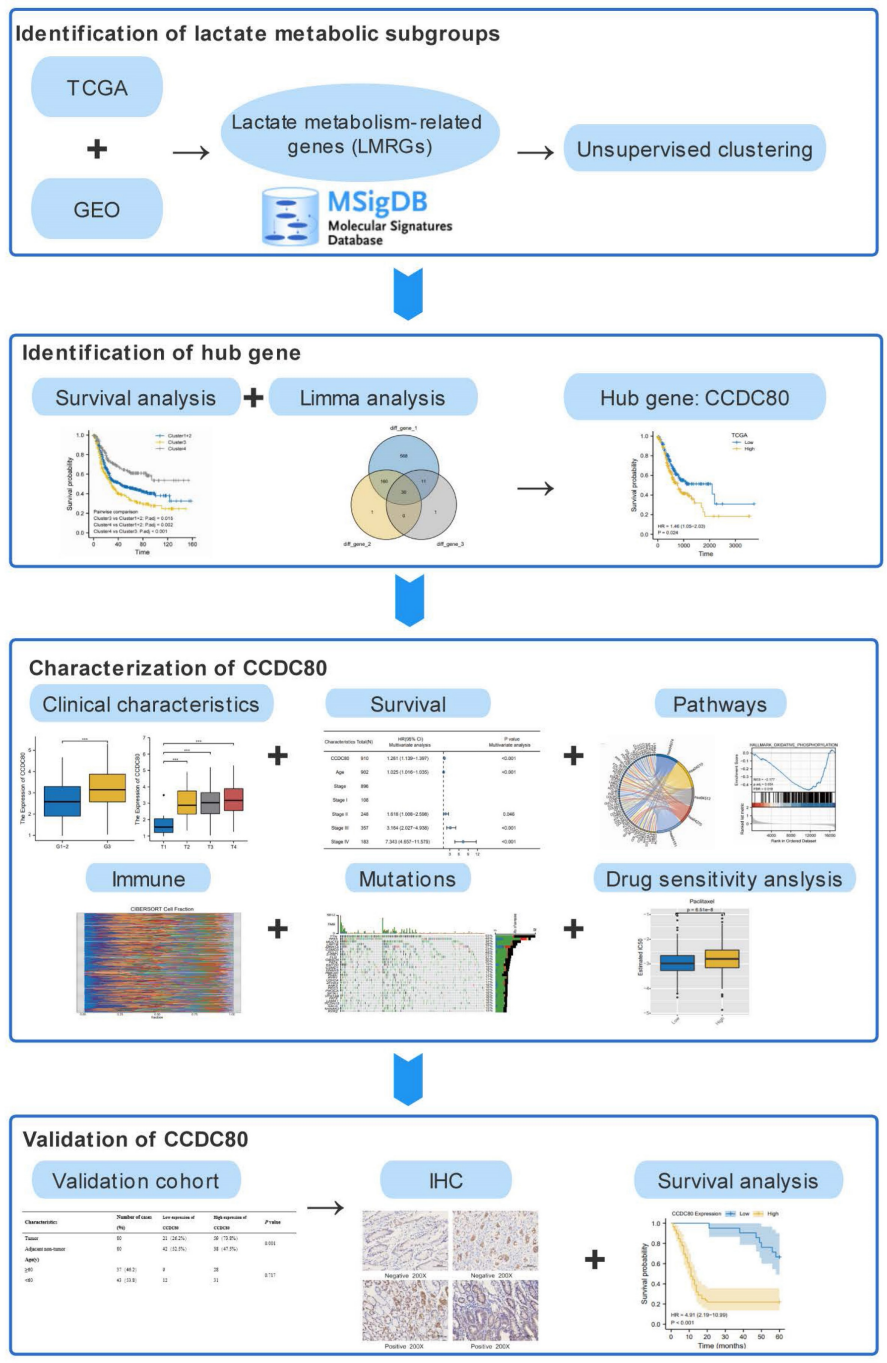
The workflow of this study.

The workflow of our study is shown in Figure 1. A total of 910 samples were included in the study, with 348 and 562 samples containing clinical and RNA sequencing data from TCGA and GEO database, respectively. The median OS time of these samples was 22 months. Of the 910 samples, 108 (12.1% of the total number of cases) were staged as Stage I, 248 (27.7% of the total number of cases) as Stage II, 357 (39.8% of the total number of cases) as Stage III, and 183 (20.4% of the total number of cases) as Stage IV. Detailed patient baseline data are presented in Table 1.

### Characterization of lactate metabolism subgroups

**Figure 2:**
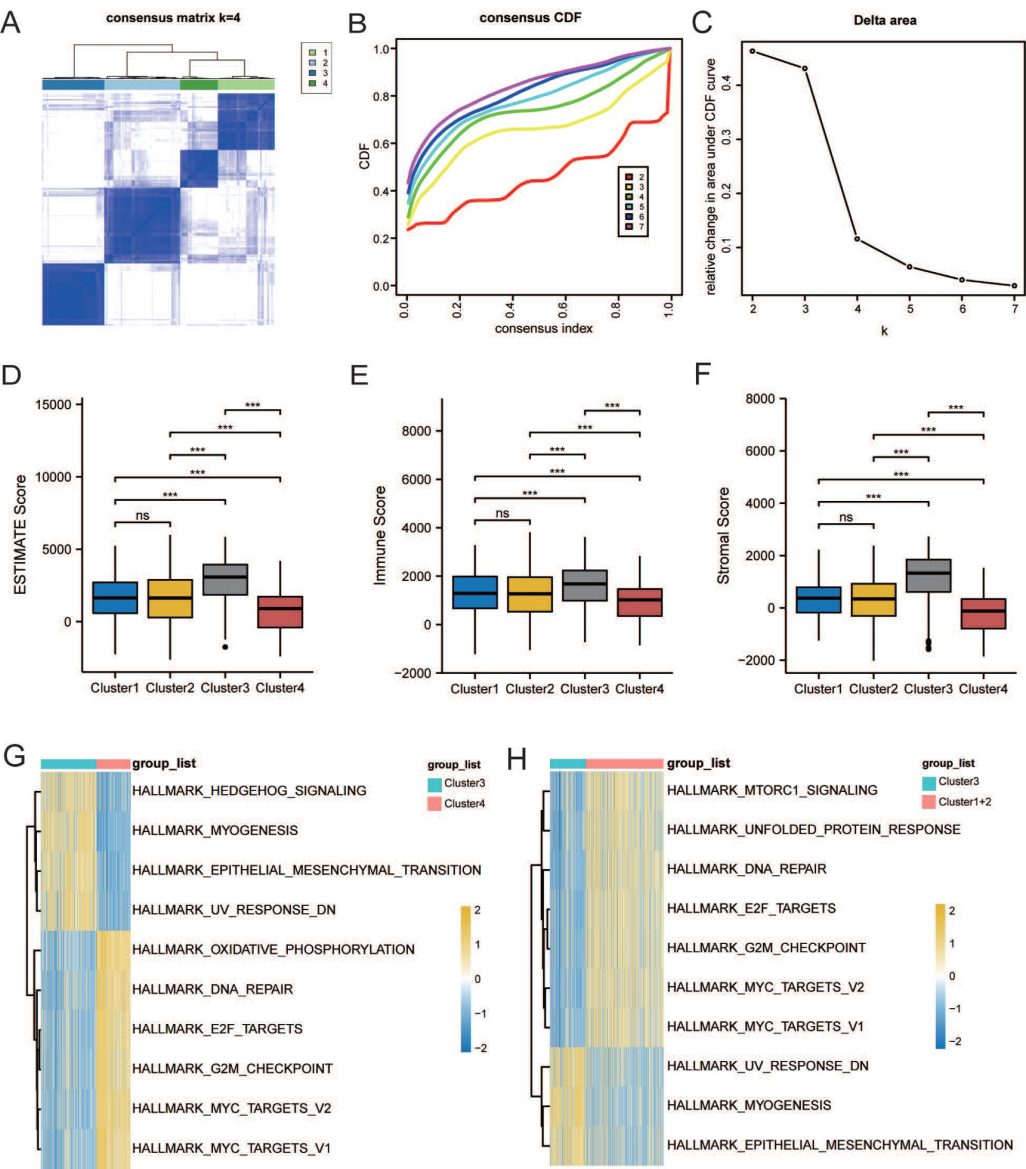
Characterization of lactate metabolism subgroups in gastric cancer (GC). **(A)** Consensus clustering matrix when k = 2. **(B)** Consensus clustering CDF with k valued 2 to 7. **(C)** Relative change in area under CDF curve. Comparisons and distributions of ESTIMATE **(D)**, immune** (E)**, and stromal scores **(F)** among clusters. Gene set variation analysis (GSVA) pathway enrichment analysis of cluster 3 and 4 **(G)**, and cluster 3 and 1+2 **(H)**.

Based on LMRG expression, we conducted unsupervised clustering analysis; we chose the clustering results at K = 4 and separated TCGA and GEO samples into four lactate metabolism subgroups (Figure 2A-C).

The ESTIMATE algorithm was used to further explore immune and stromal infiltration among these cluster subgroups. Counterintuitively, we observed no significant differences between clusters 1 and 2, but both clusters 1 and 2 had significant differences with the other two subgroups, clusters 3 and 4; an overall higher immune cell infiltration was found in cluster 3 than in the other three clusters (Figure 2D-F). As no significant differences were observed in the immune infiltration analysis between clusters 1 and 2, we subsequently combined the two groups for analysis.

Next, to further explore the function of the three subgroups, we performed GSVA. As shown in Figure 2G, the EMT pathway was significantly enriched in cluster 3, whereas the “HALLMARK_OXIDATIVE_PHOSPHORYLATION” pathway was significantly enriched in cluster 4. Thus, we further defined clusters 3 and 4 as the EMT and metabolic subtypes, respectively. As shown in Figure 2H, “HALLMARK_MTORC1_SIGNALING,” “HALLMARK_E2F_TARGETS,” and “HALLMARK_G2M_CHECKPOINT” pathways, which were related to proliferation, were enriched in cluster 1 and 2. Therefore, we further defined cluster 1+2 as the proliferation subtype.

**Figure 3:**
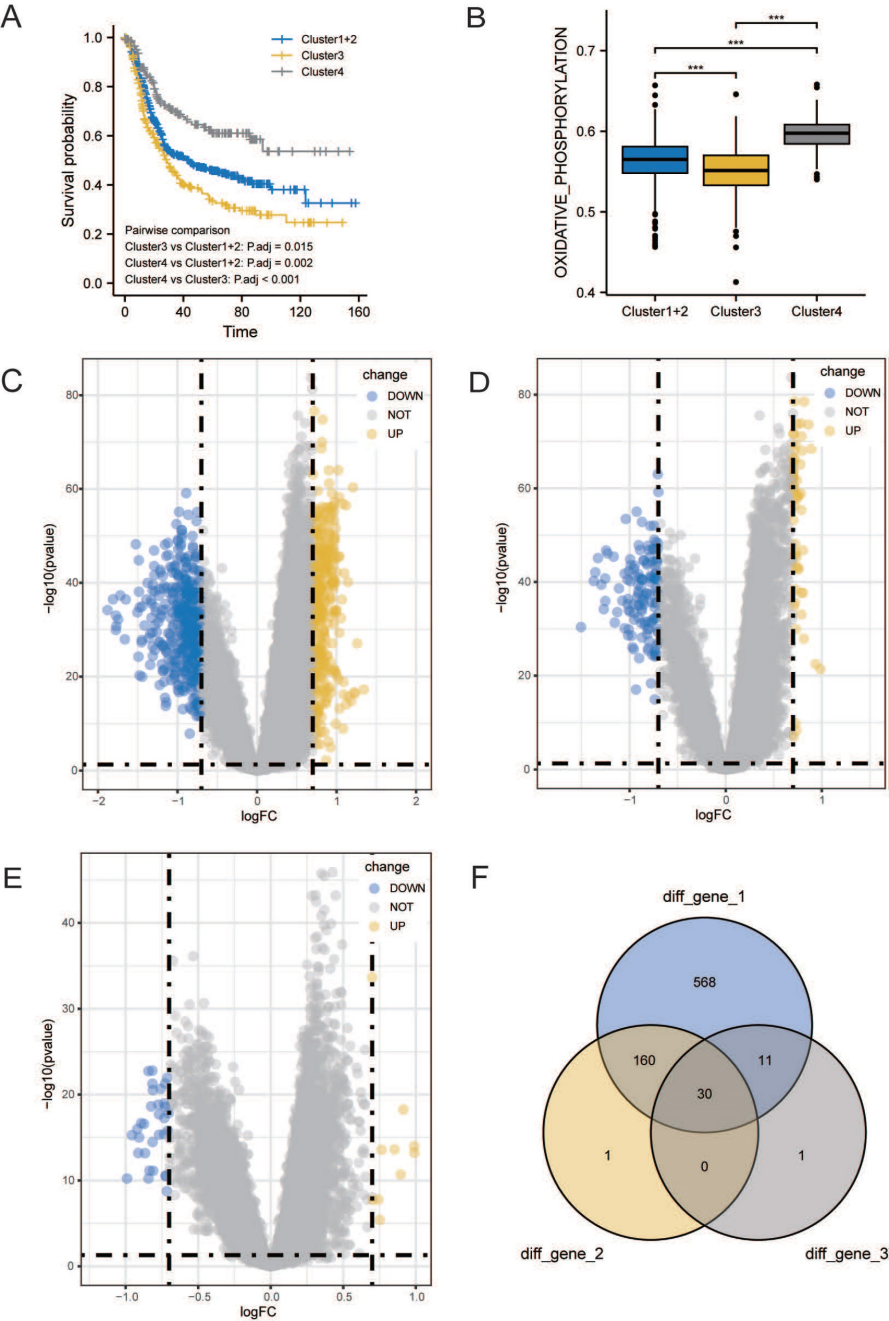
Identification of DEGs related to lactate metabolism to 7. **(A)** Overall Survival (OS) analysis of different clusters.** (B)** Comparisons of oxidative phosphorylation (OXPHOS) score among clusters. Volcano plots of DEGs between clusters 3 and 4** (C)**, clusters 3 and 1+2** (D)**, and clusters 1+2+3 and 4** (E)**.** (F)** Venn diagram indicates overlaps for differentially expressed genes (DEGs).

Survival analysis was conducted among the three subgroups. As shown in Figure 3A, OS of patients with GC statistically significantly differed among the three groups; patients in cluster 3 had the worst prognosis, whereas patients in cluster 4 had a significantly better prognosis than patients in the other two groups. We calculated the score of oxidative phosphorylation (OXPHOS) among groups using the ssGSEA algorithm; cluster 3 had the lowest score, whereas cluster 4 had the highest (Figure 3B). These results suggested that a higher OXPHOS score might be associated with a better prognosis and higher immune cell infiltration, suggesting a potential relationship between LMRGs and immune cell infiltration.

To assess the transcriptomic differences in the regulatory patterns of cellular lactate metabolism, we investigated the DEGs among different subgroups. We identified 317 up-regulated and 452 downregulated DEGs between clusters 3 and 4, 53 upregulated and 138 downregulated DEGs between clusters 3 and 1+2, and 10 upregulated and 32 downregulated DEGs between clusters 1+2+3 and 4. The volcano plots are displayed in Figure 3C-3E. The Venn diagram (Figure 3F) indicates overlaps for 30 DEGs.

In the TCGA dataset, we grouped these DEGs according to median expression and conducted a survival analysis to screen for prognostic genes with *p* < 0.05. *CCDC80* and three other genes (*PPP1R14A*, *APOD*, and *OGN*) were identified. We selected *CCDC80* for further investigation since it had not been reported with GC in the previous studies.

### *CCDC80* expression in TCGA stomach adenocarcinoma (TCGA-STAD) and Pan-Cancers

**Figure 4:**
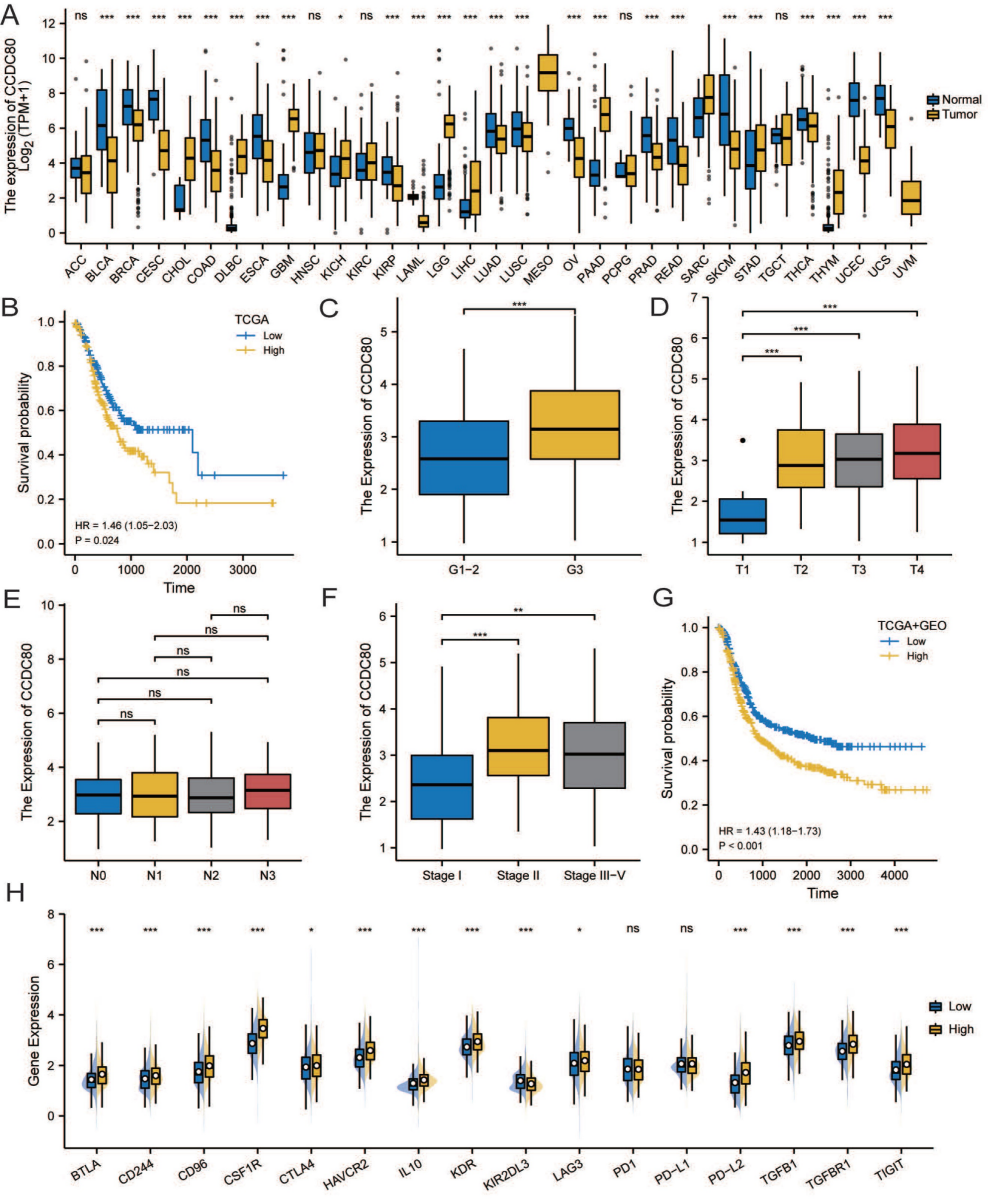
Association between Coiled-coil domain containing 80 (CCDC80) expression and different clinical characteristics. **(A)**CCDC80 expression in normal and tumor tissues in The Cancer Genome Atlas (TCGA) and Genotype-Tissue Expression (GTEx) databases. **(B)** Association between CCDC80 expression and OS in TCGA-STAD. Association between CCDC80 expression and clinical characteristics such as grade **(C)**, T stage **(D)**, N stage **(E)** and pathological stage **(F)**. **(G)** Association between CCDC80 expression and OS in TCGA and Gene Expression Omnibus (GEO) databases. **(H)** Different expressions of immune checkpoints (ICPs) in low and high CCDC80 expression groups.

We conducted a pan-cancer analysis of CCDC80 expression using data from TCGA and the Genotype-Tissue Expression (GTEx) portal. Figure 4A indicates that *CCDC80* was highly expressed in GC samples (*p* < 0.001).

In the TCGA-STAD cohort, survival analysis showed that patients with high *CCDC80* expression had lower OS than those with low expression (*p* < 0.05, Figure 4B). We further explored the relationship between *CCDC80* expression and clinicopathological variables. Figure 4C shows the relationship between *CCDC80* expression and histological grade, indicating that the *CCDC80* expression increased with a higher degree of tumor differentiation (*p* < 0.001). Similarly, *CCDC80* expression was lower in T1 (Figure 4D, *p* < 0.001) and stage I (Figure 4F,* p* < 0.05) groups. However, no significant differences were observed between the different N stages (Figure 4E).

Additionally, Figure 4G shows the prognostic value of *CCDC80* expression in the TCGA and GEO cohorts, which was consistent with the result in TCGA-STAD cohort. These results indicated that high *CCDC80* expression is a predictor of poor prognosis in patients with GC.

We then analyzed the differences in immune checkpoint (ICP) in different *CCDC80* expression groups. We confirmed that the expression of most ICPs was significantly in-creased in the high *CCDC80* expression group, which suggests that *CCDC80* expression may have an impact on immunotherapy.

### The correlation analysis of *CCDC80* and lactate metabolism

**Figure 5:**
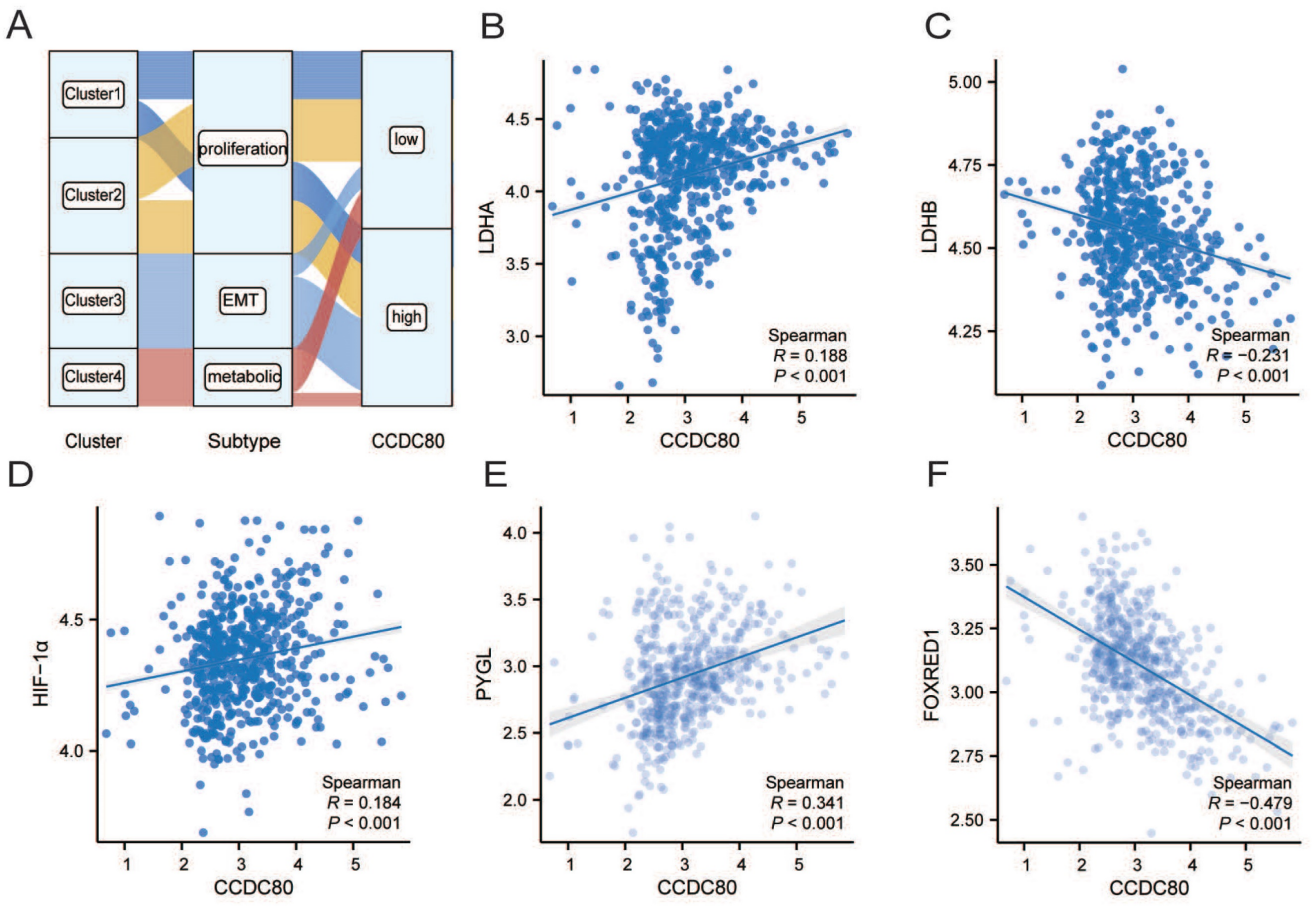
The correlation analysis of CCDC80 and lactate metabolism. **(A)**CCDC80 expression in different clusters and subtypes. Association between CCDC80 expression and LDHA **(B),** LDHB **(C)**, HIF-1α **(D)**, PYGL **(E)** and FOXRED1 **(F)**.

The analysis revealed of the patients in the metabolic subtype, most of the samples were classified in the low *CCDC80* expression group (Figure 5A). In Correlation analysis revealed a positive correlation between the mRNA expression of *CCDC80* and LDHA (Spearman's R=0.188, *p* < 0.001; Figure 5B), while a negative correlation between the mRNA expression of *CCDC80* and LDHB (Spearman's R=-0.231,* p* < 0.001; Figure 5C), although the correlations were weak. Moreover, HIF-1α expression levels were positively correlated with *CCDC80* (Spearman's R=0.184,* p* < 0.001; Figure 5D).

Further analysis of other lactate metabolism-related genes demonstrated that *CCDC80* expression was positively correlated with PYGL (Spearman's R=0.341,* p* < 0.001; Figure 5E) and negatively correlated with FOXRED1 (Spearman's R=-0.479,* p* < 0.001; Figure 5F).

### Multivariate Cox regression of *CCDC80* and nomogram model

**Figure 6:**
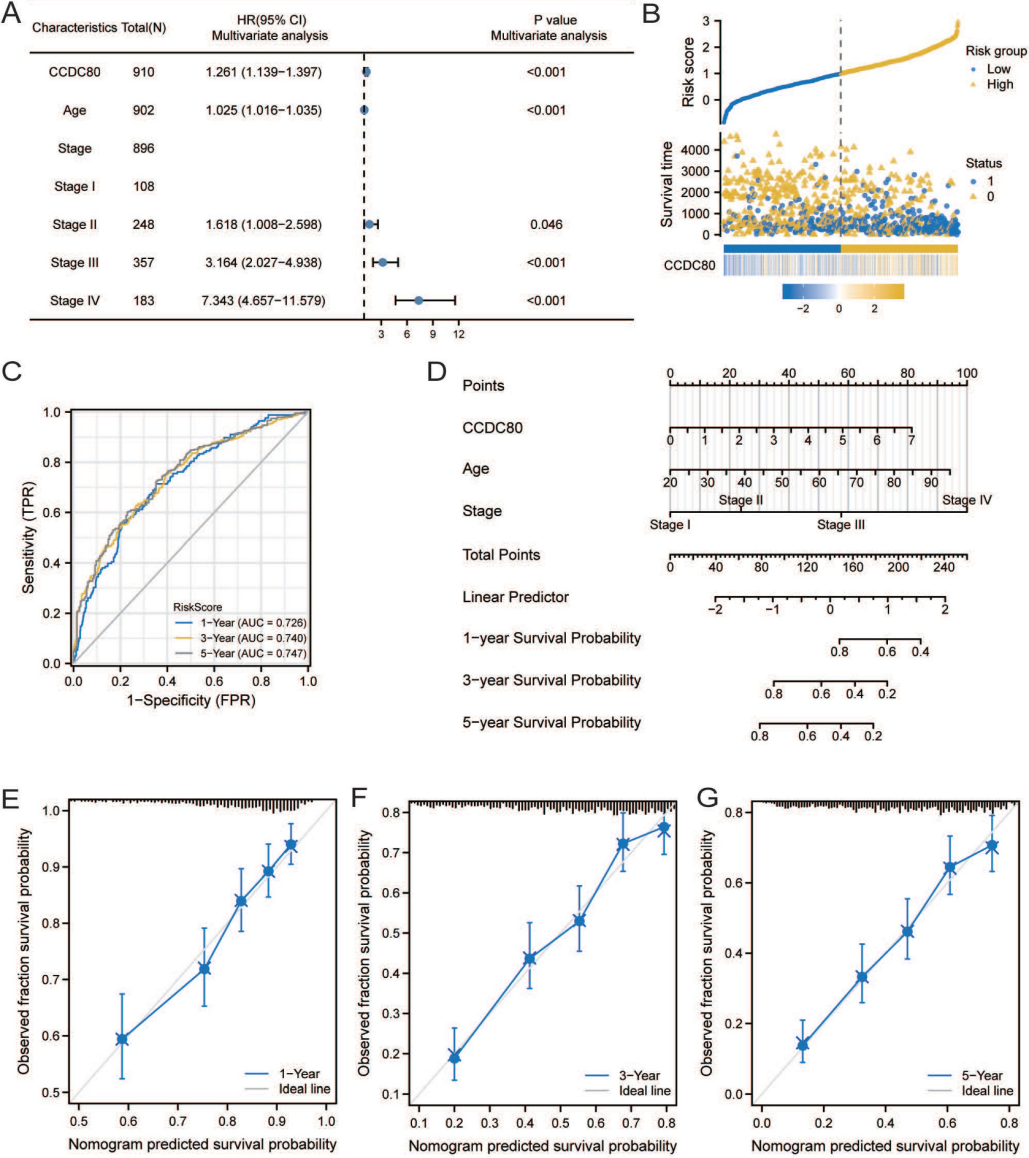
Multivariate Cox regression of CCDC80 and nomogram model. **(A)** Multivariate Cox regression for CCDC80 expression and clinical characteristics.** (B)** Risk score and survival time distributions, and heatmaps of CCDC80 expression. **(C)** ROC curve with a time dependence indicating the OS rates of 1-, 3-, and 5-year. **(D)** Nomogram integrated CCDC80 expression, age, and stage to predict OS. Calibration curves for 1- **(E)**, 3- **(F)**, and 5-year **(G)** survival predictions.

The results of univariate and multivariate Cox regression analyses are presented in Table 2. Multivariate Cox regression analysis revealed that* CCDC80* expression was an independent prognostic factor (Figure 6A). Figure 6B depicts the risk score based on multivariate Cox regression, patient survival outcomes, and *CCDC80* expression. We used the timeROC function to explore the prognosis of the risk score and then obtained the 1-, 3-, and 5-year prognostic classification efficiency. As shown in Figure 6C, the computed areas under the curve (AUC) were large: 0.726 at 1 year, 0.740 at 3 years, and 0.747 at 5 years. We constructed a nomogram to evaluate the prognosis of patients with GC. Except for *CCDC80* expression, the model included age and pathological stage (Figure 6D). Figure 6E-G shows the calibration curve for these time points.

### Pathway enrichment analysis

**Figure 7:**
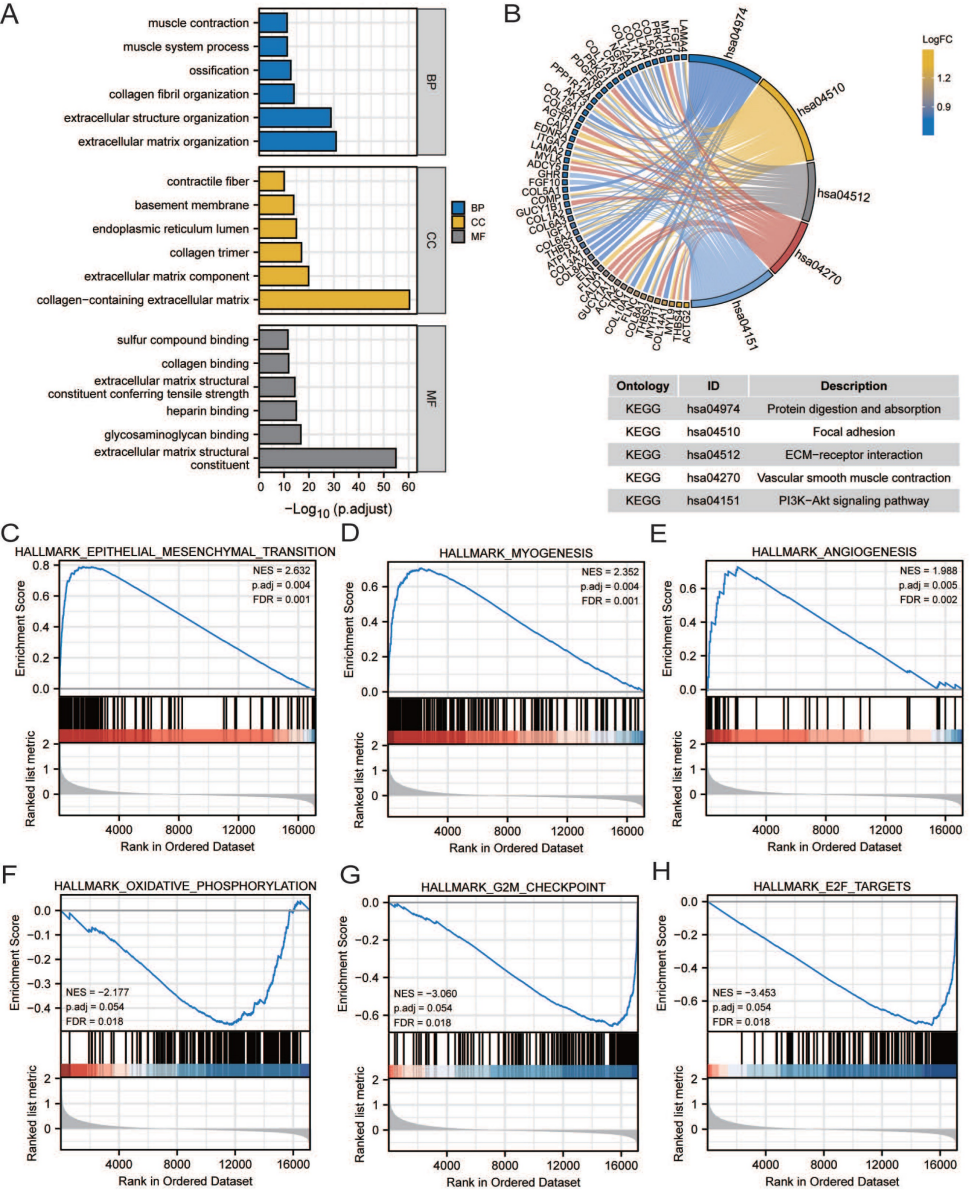
Pathway enrichment analysis. Gene ontology (GO) **(A)** and Kyoto Encyclopedia of Genes and Genomes (KEGG) **(B)** pathway enrichment of DEGs in groups with low and high CCDC80 expression. Gene set enrichment analysis (GSEA) enrichment in the high CCDC80 expression **(C-E)** and low CCDC80 expression **(F-H)**.

GO, KEGG, and GSEA analyses were performed to reveal the mechanism of *CCDC80*, using the DEGs between low and high *CCDC80* expression groups, including 387 upregulated and 13 downregulated genes. The top enriched GO terms and KEGG pathways are shown in Figures 7A and 7B. The GSEA results suggested that three functional gene sets were enriched in the high *CCDC80* expression group, which included EMT, myogenesis and angiogenesis pathways (Figure 7C-E). In contrast, OXPHOS, G2M checkpoint, and E2F targets pathways were significantly enriched in the low *CCDC80* expression group (Figure 7F-H).

### Immune cell infiltration and drug sensitivity analyses

**Figure 8:**
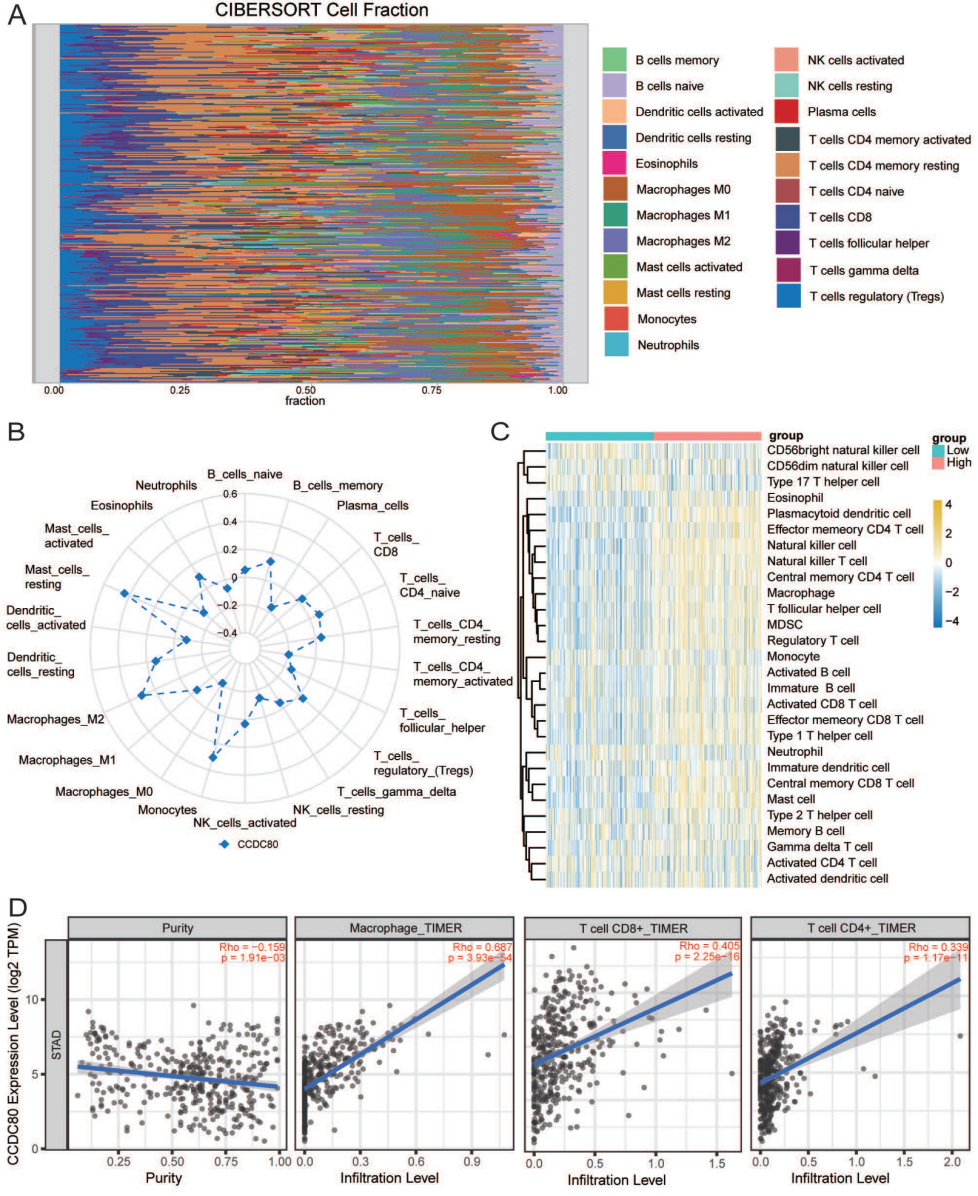
Immune infiltration analysis. **(A)** The distribution of 22 tumor infiltrating cells. **(B)** Correlation between CCDC80 expression and various immune cells. **(C)** Immune cell infiltration between low and high CCDC80 expression groups. **(D)**The correlation of CCDC80 expression with tumor purity and tumor infiltrating cells from the TIMER database.

**Figure 9:**
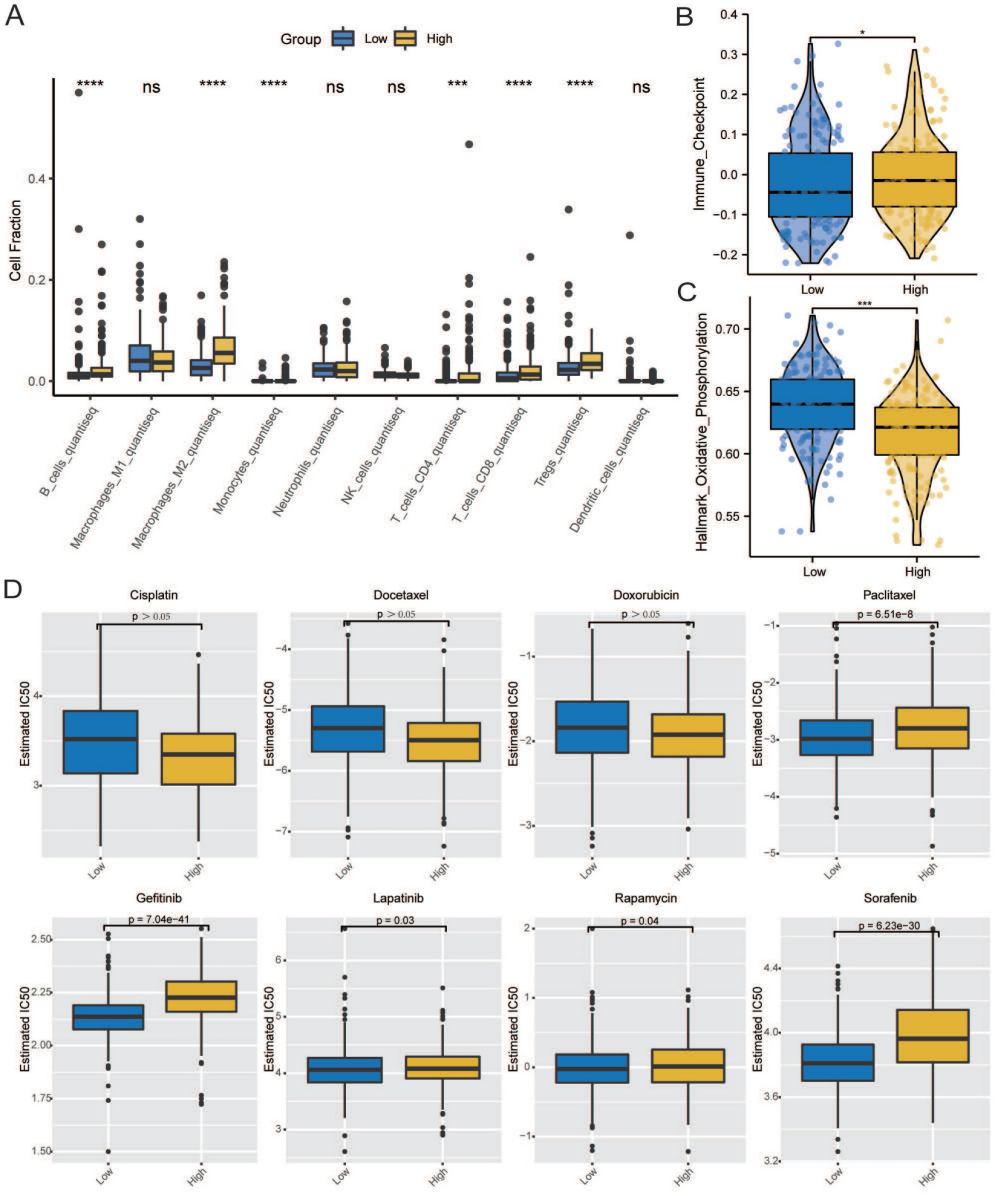
Correlation of CCDC80 expression with immunotherapy and drug sensitivity analysis. **(A)** Immune cell infiltration using quanTIseq algorithm between low and high CCDC80 expression groups. Score of ICP **(B)** and OXPHOS **(C)** in low and high CCDC80 expression groups.** (D)** Prediction of drug sensitivity.

Immune infiltration analysis was performed in the GC tumor microenvironment, as shown in Figure 8A. Figure 8B shows the correlation between *CCDC80* expression and various immune cells. This result was consistent with the TIMER database, in which the expression of *CCDC80* was significantly positively correlated with macrophage infiltration (r = 0.687, *p* < 0.001) (Figure 8D). We also compared the differences in immune cell in-filtration between the low and high *CCDC80* expression groups. As shown in Fig 8C, many immune cells were highly infiltrated in the high *CCDC80* expression group, including CD4+ T cells, CD8+ cells, B cells, and macrophages. Further analysis using the quanTIseq algorithm yielded similar results (Figure 9A). These results suggest that *CCDC80* is vital in regulating immune cell infiltration in GC. The score of important signatures was used to explore the difference in function between the high and low *CCDC80* expression groups. The high-expression group had higher ICP (Figure 9B) and lower OXPHOS scores (Figure 9C).

The results of drug sensitivity analysis can be seen in Figure 9D. It demonstrated that the drug sensitivity of paclitaxel is lower in the high *CCDC80* expression group (*p* < 0.05). In other anti-cancer drugs, such as gefitinib, lapatinib, rapamycin and sorafenib, we can see the similar results. However, we observed no significant differences in the drug sensitivity of cisplatin, docetaxel, and doxorubicin between the low and high *CCDC80* expression groups.

### Mutation characteristics

**Figure 10:**
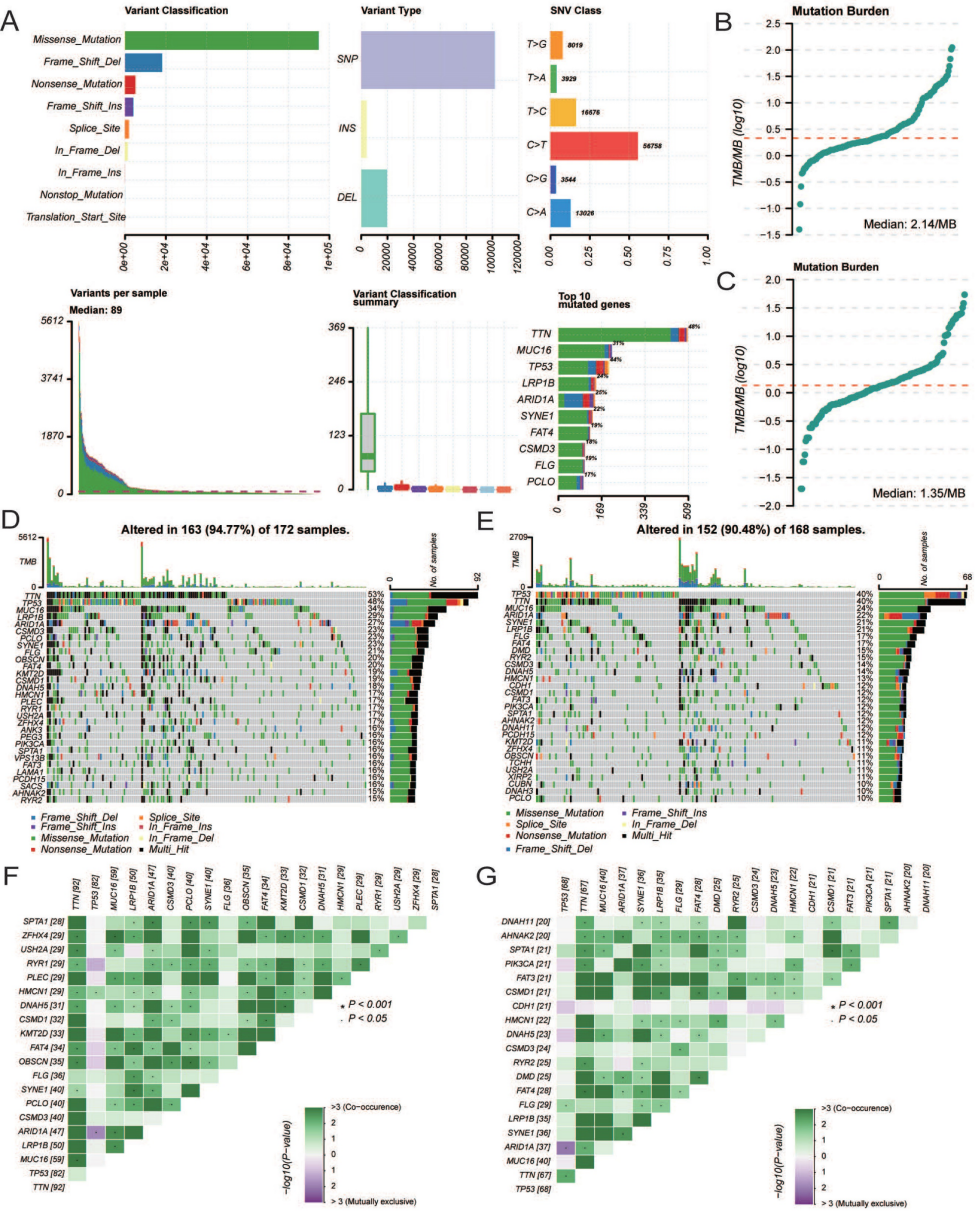
Mutation characterization in the low and high CCDC80 expression groups.** (A)** The overall mutational landscape of TCGA-STAD. Tumor mutation load (TMB) **(B, C)** and somatic mutations **(D, E)** in low and high CCDC80 expression groups. **(F, G)** The correlation between the top 20 mutated genes low and high CCDC80 expression groups.

The overall mutational landscape of TCGA-STAD is shown in Figure 10A; missense mutations occurred most frequently, and the top two mutated genes were *TTN* and *MUC16*. Subsequently, we analyzed the TMB. We observed significant differences between the groups, with the high *CCDC80* expression group exhibiting lower TMB than the low expression group (Figures 10B and 10C), indicating the impact of immunotherapy.

We analyzed the mutation characteristics of the high and low *CCDC80* expression groups. The top 30 mutated genes in the low and high *CCDC80* expression groups were mapped (Figures 10D and 10E). *TTN* and *MUC16* were more frequently mutated in the low *CCDC80* expression group, with mutation frequencies of 53% and 34%, respectively. Figure 10F and 10G shows the correlation between the top 20 mutated genes. These results provide novel insights into the intrinsic connection between immunotherapy and somatic variation.

### CCDC80 expression in our GC samples and its relation with clinicopathological characteristics

**Figure 11:**
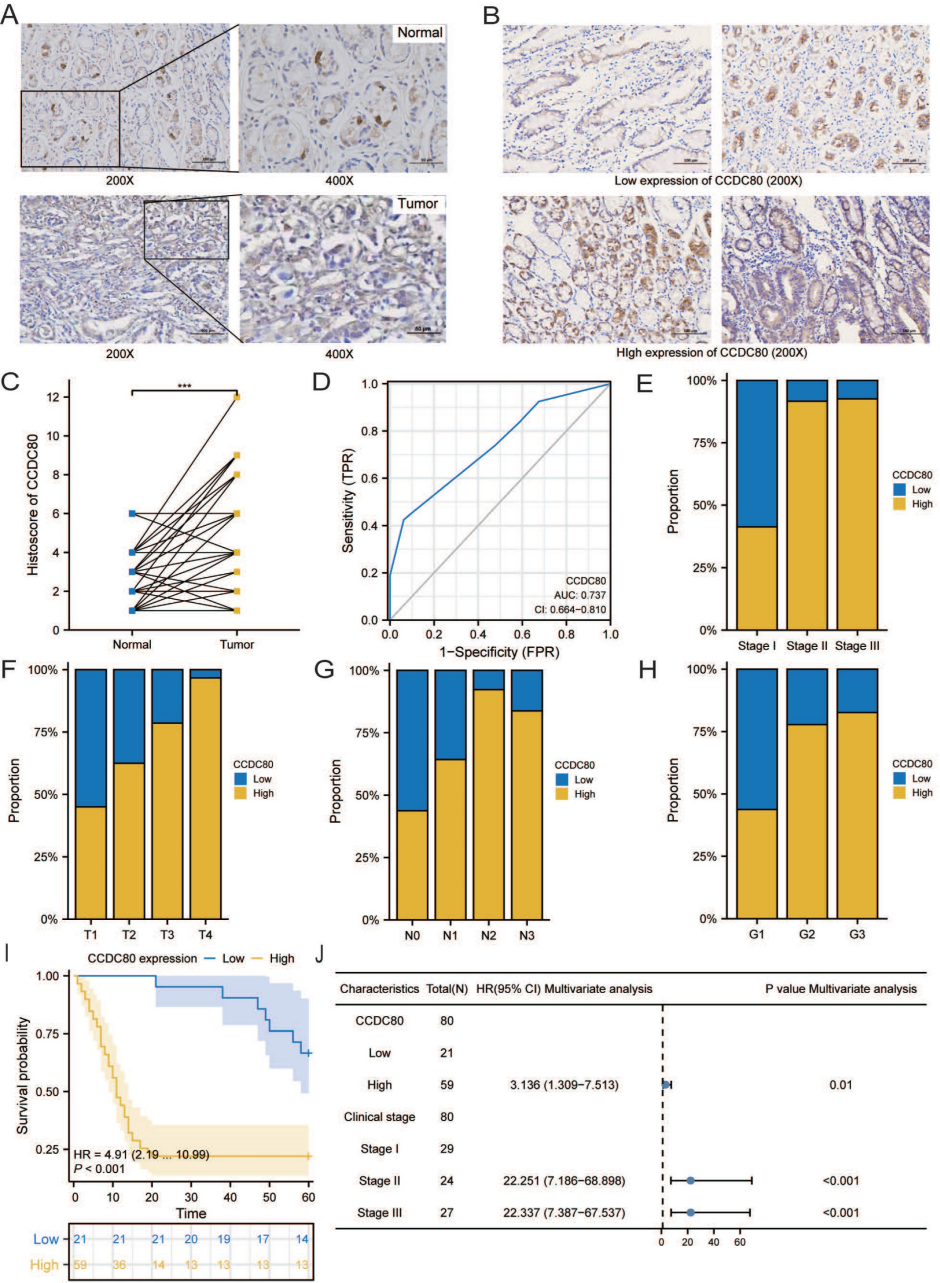
Immunohistochemistry and clinical analysis of CCDC80 in GC. **(A)** Representative Immunohistochemistry image of CCDC80 and subcellular staining localization in GC and adjacent non-tumor tissue specimens. **(B)** The representative staining of low and high CCDC80 expression. **(C)** The expression of CCDC80 in GC tissue was higher than adjacent non-tumor tissue (*p* = 0.001). **(D)** Receiver-operator characteristics curve. Comparison of clinical stage **(E)**, T stage** (F)**, N stage **(G)** and grade **(H)** in low and high CCDC80 expression groups. **(I)** OS analysis revealed that high CCDC80 expression indicates a poor prognosis (*p* <0.001). **(J)** The forest plot of multivariate Cox regression.

The age of the independent cohort was 28-80 years old (≥60 years old, n = 37, 46.2%; <60 years old, n = 43, 53.8%), and the cohort included both male (n = 50, 62.5%) and female (n = 30, 37.5%) (Table 3). Patients with clinical stages I (n = 29, 36.3%), II (n = 24, 30%), and III (n = 27, 33.7%) were present. Both GC and adjacent non-tumor tissue specimens exhibited cytoplasmic CCDC80 expression. CCDC80 staining was significantly more intense in GC tissue specimens (score ≥4, 73.8% [59/80]) than in adjacent non-tumor tissue specimens (score ≥4, 47.5% [38/80]) (*p* = 0.001) (Figure 11A-C). High CCDC80 protein expression was positively correlated with the clinical stage (*p* < 0.001), T stage (*p* < 0.001), N stage (*p* = 0.004), and pathologic differentiation (*p* = 0.009). Regarding the ability of CCDC80 expression to discriminate between patients with GC and healthy individuals, the ROC area under the curve was 0.737 (Figure 11D).

Subsequently, we analyzed the distribution of different clinical characteristics in the high and low CCDC80 expression groups. As shown in Figure 11E-H, the proportion of patients with Stage I in the high CCDC80 expression group was lower than 50%, whereas that of patients with Stages II and III was approximately 90%. Furthermore, we found that CCDC80 expression was higher in patients with a higher degree of malignancy. Survival analysis based on the histoscore of CCDC80 indicated that patients with relatively high CCDC80 expression had poorer OS than those with low CCDC80 expression (*p* < 0.001) (Figure 11I). Multivariate Cox regression indicated that clinical stage (*p* < 0.001) and CCDC80 expression level (HR = 3.316; 95% CI [1.309-7.531]; *p* = 0.01) were prognostic factors independently correlated with poor OS (Figure 11J; Table 4).

## Discussion

Gastric cancer (GC) is one of the most common and deadly malignancies worldwide, significantly impacting human health and quality of life. The high mortality rate is primarily due to late-stage diagnosis and limited effective treatment options^36^. Therefore, studying the biological behavior and molecular mechanisms of GC is of considerable significance for accurately predicting prognosis and screening novel targets of GC.

This study analyzed GC data from TCGA and GEO databases using unsupervised clustering of LMRGs and defined three subgroups, and then we focused on the *CCDC80* gene and its relationship with immune cell infiltration, drug sensitivity, and clinical prognosis in GC. Previous research has highlighted the role of metabolic reprogramming and immune microenvironment in cancer progression and treatment response^7^, ^37^. Our research aims to uncover potential mechanisms and therapeutic targets that could enhance the diagnosis and treatment of GC, offering promising avenues for personalized medicine and improved clinical outcomes.

*CCDC80* expression is closely related to patient prognosis in various malignant tumors. For example, overexpression of* CCDC80* suppresses epithelial-mesenchymal transition (EMT) and cell migration in pancreatic cancer^15^. Additionally, in colorectal cancer, *CCDC80* acted as a suppressor of tumor growth, with research indicating that it involved the phosphorylation of ERK^14^. However, in our study, high* CCDC80* expression is associated with poor overall survival (OS) in GC patients, suggesting that *CCDC80* may play different roles in various types of tumors. The correlation analysis between* CCDC80* and clinical pathological parameters showed that *CCDC80* expression was related to T and pathological stages. However, the expression of *CCDC80* did not significantly differ among the different N stages. Therefore, we hypothesized that *CCDC80* might affect the prognosis of GC by affecting tumor growth.

Additionally, *CCDC80*'s positive correlation with LDHA and negative correlation with LDHB, although weak, implies its involvement in lactate metabolism, potentially influencing the tumor microenvironment and cancer cell metabolism. LDHA is a key enzyme in the glycolytic pathway, catalyzing the conversion of pyruvate to lactate. LDHB is another isoform of lactate dehydrogenase that preferentially converts lactate to pyruvate, playing a crucial role in lactate metabolism^38^. Understanding the interplay among *CCDC80*, LDHA and LDHB could provide insights into the metabolic reprogramming of cancer cells and identify novel targets for therapeutic intervention aimed at restoring normal metabolic processes and enhancing anti-tumor immunity.

Glycogen phosphorylase L (PYGL) is a key enzyme in glycogen metabolism, which is upregulated under hypoxic conditions through the HIF-1α pathway, thereby promoting glycogen breakdown and glycolysis, providing energy support for cells in hypoxic environments^39^. FAD-dependent oxidoreductase domain containing 1 (FOXRED1) is an important enzyme involved in mitochondrial function and redox reactions. It can regulate the activity of mitochondrial respiratory chain complex I, thereby affecting cellular energy metabolism and oxidative stress response^40^. The correlation between *CCDC80* and PYGL and FOXRED1 is stronger, which may suggest that CCDC80's impact on lactate metabolism may occur through multiple pathways, including glycogen metabolism and mitochondrial function.

To further explore the mechanisms of *CCDC80* in GC, we applied GSEA to identify pathways enriched in *CCDC80*; the OXPHOS pathway and angiogenesis were identified in the low and high *CCDC80* expression groups, respectively. Based on this, we speculated that *CCDC80* overexpression might promote the Warburg effect and inhibit OXPHOS in tumor cells, leading to the production of more lactate, which promotes angiogenesis 41.

The gene's role in modulating immune checkpoints further underscores its importance, as increased expression of immune checkpoint molecules in the high CCDC80 expression group indicates a possible impact on immune evasion mechanisms. The tumor microenvironment (TME), which includes tumor cells, stromal cells, and multiple types of immune cells, plays a vital role in tumor progression and metastasis. In the tumor microenvironment, immune cells are inclined to promote tumor growth instead of exerting antitumor effects 42. We further analyzed the relationship between *CCDC80* and immune cell infiltration and observed that *CCDC80* expression was significantly correlated with the infiltration of various immune cells, including mast cells, monocytes, and M2 macrophages. Lactate can promote macrophage polarization towards the M2 subtype 43. Notably, the high *CCDC80* expression group had statistically significantly higher levels of M2 macrophage infiltration than the low expression group. Therefore, high *CCDC80* expression may potentially affect the TME through M2 macrophage polarization, which could promote tumor progression 44-46. The dual role in immune modulation and metabolic regulation positions *CCDC80* as a critical player in GC pathogenesis and a potential target for therapeutic intervention.

Paclitaxel is a common chemotherapy drug that exerts its function by blocking mitosis. However, paclitaxel resistance remains a crucial issue that requires resolution 47. Our results revealed that high *CCDC80* expression is related to decreased sensitivity to paclitaxel. However, no significant differences were found for cisplatin. The observed resistance to chemotherapeutic agents such as paclitaxel in high *CCDC80* expression groups further highlights the need for targeted therapies that can overcome this resistance and improve patient outcomes. These findings may have significant consequences for personalized medicine.

Finally, we explored the mutation status of cancer-related genes, which may have an impact on treatment strategies 48. *TTN* and *MUC16* mutations are associated with GC prognosis 49. In our study, the low *CCDC80* expression group tended to have higher *TTN* and *MUC16* mutation frequencies. The TMB was lower in the high *CCDC80* expression group than in the low expression group. This was consistent with previous reports that patients with a higher TMB had better survival outcomes 50. The lower TMB observed in high CCDC80 expression groups could imply a reduced likelihood of benefiting from such therapies, further emphasizing the need for personalized treatment strategies.

However, our study has the following limitations: First, the sample size, particularly for the clinical validation cohort from the First Hospital of China Medical University, is relatively small, which may limit the generalizability of the findings. Additionally, while we have corrected for batch effects, the use of multiple datasets from different sources (TCGA and GEO) could still introduce variability that may affect the results. Finally, the lack of extensive clinical validation and functional assays means that the clinical applicability of CCDC80 as a prognostic marker or therapeutic target in gastric cancer remains to be fully established.

In conclusion, our findings divided patients with GC into proliferation, EMT, and metabolic subtypes, and revealed that *CCDC80* is a poor prognostic factor in GC and that *CCDC80* may affect the prognosis of GC by regulating OXPHOS and lactate metabolism. Patients with higher *CCDC80* expression tended to have drug resistance to paclitaxel. These results might help to guide and personalize patient anticancer treatment decisions.

## Figures and Tables

**Table 1:** Clinical information of the 910 samples used in this study.

Characteristics	Number of cases (%)	TCGA	GEO
n	910	348	562
			
Age, median (IQR)	65 (56.76, 72)	67.35 (58.48, 73.15)	64.35 (56, 70.12)
			
Gender			
Male	601 (66%)	225	376
Female	309 (34%)	123	186
			
Stage			
Stage I	108 (11.8%)	46	62
Stage II	248 (27.3%)	110	138
Stage III	357 (39.2%)	144	213
Stage IV	183 (20.1%)	34	149
NA	14 (1.5%)	14	0
			
Status			
Alive	482 (53%)	203	279
Dead	428 (47%)	145	283
			
OS time (months), median (IQR)	22 (11.128, 58)	15.48 (9.2, 26.16)	32.67 (12.82, 70.57)

**Table 2:** Results of univariate and multivariable analysis.

Characteristics	Total (N)	Univariate analysis			Multivariate analysis	
		Hazard ratio (95% CI)	P value		Hazard ratio (95% CI)	P value
**CCDC80**	910	1.244 (1.132-1.368)	**<0.001**		1.261 (1.139-1.397)	**<0.001**
						
**Age**	902	1.014 (1.005-1.022)	**0.003**		1.025 (1.016-1.035)	**<0.001**
						
**Gender**	910					
Male	601	Reference				
Female	309	0.844 (0.688-1.037)	0.106			
						
**Stage**	896					
Stage I	108	Reference				
Stage II	248	1.670 (1.041-2.679)	**0.034**		1.618 (1.008-2.598)	**0.046**
Stage III	357	3.291 (2.113-5.126)	**<0.001**		3.164 (2.027-4.938)	**<0.001**
Stage IV	183	6.766 (4.304-10.637)	**<0.001**		7.343 (4.657-11.579)	**<0.001**

**Table 3:** Clinical characteristics of patients from The First Hospital of China Medical University and correlations between CCDC80 expression and clinicopathological characteristics.

Characteristics	Number of cases (%)	Low expression of CCDC80	High expression of CCDC80	*P* value
Tumor	80	21 (26.2%)	59 (73.8%)	0.001
Adjacent non-tumor	80	42 (52.5%)	38 (47.5%)	
Age(y)				
≥60	37 (46.2)	9	28	0.717
<60	43 (53.8)	12	31	
Gender				
Male	50 (62.5)	12	38	0.555
Female	30 (37.5)	9	21	
Clinical stage				
Stage I	29 (36.3)	17	12	<0.001
Stage II	24 (30.0)	2	22	
Stage III	27 (33.7)	2	25	
T stage				
T1	20 (25)	11	9	<0.001
T2	16 (20)	6	10	
T3	14 (17.5)	3	11	
T4	30 (37.5)	1	29	
N stage				
N0	16 (20)	9	7	0.004
N1	14 (17.5)	5	9	
N2	13 (16.3)	1	12	
N3	37 (46.2)	6	31	
Pathologic differentiation				
G1	16 (20.0)	9	7	0.009
G2	18 (22.5)	4	14	
G3	46 (57.5)	8	38	
Histological type				
Papillary type	4 (5.0)	1	3	0.995
Tubular type	27 (33.7)	7	20	
Poorly differentiated type	24 (30.0)	7	17	
Signet Ring type	9 (11.3)	2	7	
Mucinous type	16 (20.0)	4	12	
Venous invasion				
No	70 (87.5)	17	53	0.501
Yes	10 (12.5)	4	6	
Lymphatic invasion				
No	56 (70.0)	16	40	0.471
Yes	24 (30.0)	5	19	

**Table 4:** Univariate and multivariate Cox regression analyses incorporating clinicopathological characteristics of patients from The First Hospital of China Medical University.

Characteristics	Total(N)	Univariate analysis			Multivariate analysis	
		Hazard ratio (95% CI)	P value		Hazard ratio (95% CI)	P value
**CCDC80**	80					
Low	21	Reference				
High	59	4.910 (2.194-10.989)	**<0.001**		3.136 (1.309-7.513)	**0.010**
						
**Age**	80					
<60	43	Reference				
≥60	37	0.910 (0.530-1.564)	0.733			
						
**Clinical stage**	80					
Stage I	29	Reference				
Stage II	24	23.788 (7.942-71.253)	**<0.001**		22.251 (7.186-68.898)	**<0.001**
Stage III	27	25.072 (8.448-74.408)	**<0.001**		22.337 (7.387-67.537)	**<0.001**
						
**Pathologic differentiation**	80					
G1	16	Reference				
G2	18	1.679 (0.716-3.940)	0.234			
G3	46	1.553 (0.738-3.271)	0.246			
						
**Venous invasion**	80					
No	70	Reference				
Yes	10	1.236 (0.583-2.623)	0.581			
						
**Lymphatic invasion**	80					
No	56	Reference				
Yes	24	0.737 (0.400-1.359)	0.328			
